# ParentingWell: adapting a family-focused practice for parents with mental illness

**DOI:** 10.3389/fpsyt.2025.1678134

**Published:** 2025-09-19

**Authors:** Joanne Nicholson, Miriam Heyman, Fernanda Escobar, Katharine Kaplan

**Affiliations:** ^1^ Heller School for Social Policy and Management, Brandeis University, Waltham, MA, United States; ^2^ Biden School of Public Policy and Administration, University of Delaware, Newark, DE, United States

**Keywords:** parents with mental illness, family-focused practice, intervention adaptation, behavioral health services, implementation evaluation

## Abstract

**Introduction:**

The ParentingWell Practice Approach is a family-focused practice approach for adults who are parents receiving mental health services. The ParentingWell Learning Collaborative (PWLC) was originally developed and tested within the Massachusetts behavioral health service system to prepare and support mental health practitioners in implementing ParentingWell. The purpose of the current study was to systematically adapt ParentingWell, including the PWLC, for further implementation and scaling-out in a new setting with a diverse target population, and address the following question: What are the essential considerations in adapting ParentingWell resources to a diverse, vulnerable, at-risk target population in an urban service delivery context?

**Methods:**

We used a participatory approach, developmental evaluation design and mixed methods to document the adaptation process, and to assess preliminary acceptability, fit, and feasibility. The adaptation process included (1) establishment of an Adaptation Team consisting of a diverse and multi-disciplinary team of policy makers and practitioners; (2) review of ParentingWell content by community stakeholders; and (3) piloting of the PWLC model in the new context, with local agency personnel.

**Results:**

The Adaptation Team provided guidance related to enhancing the acceptability of ParentingWell and the PWLC, including considerations related to the training format and evaluation methods. Community stakeholders provided suggestions to strengthen the fit of ParentingWell resources, including the creation of plain language resources. Data from PWLC participants indicated that they benefitted from participation in the Learning Collaborative.

**Discussion:**

This study provides preliminary evidence for the acceptability, fit, and feasibility of ParentingWell in an urban service context. Future research should include longitudinal data collection with both providers and parents to identify how providers use ParentingWell tools and strategies, and to evaluate the impact of ParentingWell on parents served and their children.

## 1 Introduction

### 1.1 Parents with mental illness and their families

Nearly one-quarter of American parents with minor children (under the age of 18) living in their households had a mental illness in the past year; nearly 6% had a serious mental illness (1). Adults with mental illnesses are as likely as their counterparts without mental illness to be parents (2). The impact of mental health conditions on both adults who are parents and their children are well documented (3, 4). The issues in providing family-focused practice, limitations in interventions and intervention research, and challenges to sustainability have been described, including stigma, providers’ attitudes and skills, organizational capacity, shifts in policies or mandates, and the challenges in integrating relevant outcome measures into routine workflow (5–11). Several models with growing evidence bases have emerged, including Family Talk (12–14), the Family Model (15–17), Child Talks (18–21), and Let’s Talk about Children (22–25).

The focus of the current study is the adaptation of a family-focused practice, ParentingWell, in a new service context, Philadelphia, Pennsylvania, with a diverse target population. ParentingWell, drawn from the evidence-based Let’s Talk about Children (LTC) intervention, was originally adapted for adults who are parents receiving mental health services in Massachusetts, described in a previous publication (26). In this prior publication we described LTC in detail, the selection of LTC, and the rationale for adaptation in Massachusetts. The previous adaptation effort involved international experts and Massachusetts stakeholders, including individuals with lived experience, peer specialists, clinicians, case managers, and advocates, representing diverse geographic areas across Massachusetts. Implementation challenges identified in Massachusetts informed the original adaptation and the compilation of ParentingWell, while retaining the core elements and practice principles derived from LTC. Based on information obtained from stakeholders, we determined that practitioners needed and wanted a practice approach with well-specified core elements and principles, that fit into their routine workflow, drew from existing skills and competencies, and could be used in working with parents of any age, in the context of on-going therapeutic relationships.

In the current manuscript, we describe the process of adapting ParentingWell in Philadelphia, a metropolitan setting with an extremely diverse target population, which differs substantially from the Massachusetts adaptation setting (i.e., a state-wide geographic region with a significantly less diverse population). In particular, Philadelphia is home to immigrants from Asia, Latin America, the Caribbean, Europe, and Africa, with Black or Hispanic, economically-challenged individuals comprising the largest portion of individuals receiving public sector behavioral health services (27). While many of the provider, organizational, and systems level challenges identified in Massachusetts are similar in the Philadelphia service context (e.g., the lack of integration of adult and child services to promote family-focused practice), the support of the service payer and the close network of relationships among Philadelphia services suggested new considerations and provided additional opportunities for adaptation and implementation.

### 1.2 The adaptation process

The process of the adaptation of evidence-based interventions for “scaling-up” and “scaling-out” in new contexts, with new target populations has received increased attention, set in the context of implementation science (28–33). Effective intervention adaptation, at best, requires initial specification of intervention core elements and core functions, with efforts made to maintain fidelity to the original model, as fit and feasibility are assessed in the new setting (31). Acceptability, fidelity, and feasibility are context and population dependent (34). While steps in intervention adaptation have generally been described (e.g., exploration, preparation, implementation, and sustainment), the process is considered to be iterative and dynamic (29, 31–33). Authors have highlighted the benefits of engaging diverse stakeholders as key partners (e.g., persons served, practitioners, agency administrators, community members) in the adaptation process (29, 30), with stakeholders’ perceptions likely to influence implementation and uptake (34).

In the exploration step or phase, intervention key ingredients, action mechanisms, activities, and best practices are identified, comprising the intervention logic model (28, 29, 32). Next, essential modifications in intervention model and materials are prepared, to optimize the fit and feasibility of the adapted model for the new context, informed by feedback from stakeholders in the new service setting (33). The third phase, implementation, involves pilot testing of the adapted model, with training of staff, to inform and further refine model adaptations. In the final phase, sustainment, the intervention is implemented and evaluated further. Training and ongoing support may enhance intervention results (35).

### 1.3 The ParentingWell practice profile and learning collaborative

The ParentingWell Practice Profile is the result of the initial process of adapting the evidence-based Let’s Talk about Children (LTC) intervention for use in Massachusetts (26). To address the need for family-focused practice with parents with mental illnesses, a number of potential interventions were considered. LTC, developed in Finland (24) and replicated and tested in Australia (22, 36–38), Greece (39), China (40), and Japan (41), comprised the evidence based intervention for adaptation in Massachusetts. LTC is a structured intervention in which trained practitioners incorporate conversations about children during their interactions with adult clients who are parents. The intervention includes opportunities for the practitioner and client to discuss children, parental mental illness and its impact on family life, pathways to enhance family and child wellbeing, and available resources to support the family (42).

ParentingWell was adapted and implemented in collaboration with Massachusetts mental health service vendors, in partnership with the Massachusetts Department of Mental Health (26). The initial adaptation process took place between 2015 and 2019 and included the following steps: (1) identifying the core elements and key theories underlying LTC, in collaboration with the creators of the original intervention; (2) working with key stakeholders in the Massachusetts behavioral health system to identify relevant aspects of the service context and target population, including client characteristics; (3) pretesting preliminary adapted content; and (4) refining the adapted content based on stakeholder feedback.

In the Massachusetts adaptation initiative, 70 community stakeholders with professional and lived experience participated in facilitated discussions about: (1) the experiences of parents and practitioners; (2) services currently provided; (3) challenges and unmet needs; and (4) implementation issues, current or anticipated, related to community and agency contexts, and the characteristics of the workforce and persons served (26). Identified implementation challenges included aspects at the individual provider level (e.g., attitudes, beliefs, skills), organizational level (e.g., agency culture, workflow and routines, paperwork gaps, crisis orientation, lack of referral resources), and systems level (e.g., policy gaps, mandate misunderstandings).

Adaptation efforts in Massachusetts yielded the ParentingWell Practice Profile, which retains the core elements and key principles of LTC, and can be integrated into routine interactions between practitioners and persons served (43). ParentingWell includes tools and conversation prompts that prepare practitioners to engage in family-focused conversations, develop family-informed service plans, and generally support adults in a behavioral health setting while consistently integrating conversations around parenting and family life (44). The ParentingWell Practice Profile is meant for use by diverse practitioners in multiple settings; reflecting considerations related to parenting across the life span; and drawing from and building on practitioners’ existing skills and knowledge. The four core elements align with Self-Determination Theory (engage, explore, plan, access and advocate) and four principles are consistent with LTC (trauma-informed, strengths-based, family-focused, culturally sensitive) (26).

The ParentingWell Learning Collaborative (PWLC) was implemented in Massachusetts to prepare and support mental health practitioners in implementing ParentingWell and provided opportunity to pretest the adapted content and pilot training (45). The Massachusetts PWLC included in-person orientation, training and debriefing sessions, virtual coaching sessions, and an interactive online platform for dialogue and resource sharing. Participants in the initial PWLC were highly engaged in activities and satisfied with learning opportunities. They reported active implementation of ParentingWell skills, tools, and resources (45, 46). Modifications were made to final intervention resources, incorporating feedback from PWLC participants. While there is preliminary evidence supporting the utility of the PWLC in the Massachusetts service context, further characteristics of persons served, intervention content, and contextual considerations are key to the intervention adaptation process for use and testing in other contexts (47).

### 1.4 The study purpose

The purpose of this intervention adaptation study was to systematically adapt ParentingWell for further implementation and scaling-out in a new setting with a diverse target population (28, 31, 33). We addressed the following question: What are the essential considerations in adapting the ParentingWell resources to a diverse, vulnerable, at-risk target population in a new, urban service delivery context? A planned, proactive process was carried out with diverse community stakeholders to: (1) explore community characteristics, available resources, and the needs of persons served (context); (2) review and prepare ParentingWell materials to be relevant in the new community setting (content); and (3) trial the adapted materials with diverse practitioners (training) (48). Acceptability, fit, and feasibility were assessed throughout the dynamic, iterative adaptation process. The goal of this paper is to describe the adaptation process as the first step in implementing and sustaining ParentingWell in a new community context, with a new, diverse target population.

## 2 Methods and procedures

In the current study, a participatory approach, developmental evaluation design and mixed methods were employed to document the adaptation process regarding intervention context, content, training and evaluation; and assess preliminary acceptability, fit and feasibility (33, 34, 48, 49). Overlapping phases of the adaptation process included the establishment of an Adaptation Team, ParentingWell content review by community stakeholders, and piloting of the ParentingWell Learning Collaborative model in the new context, with local agency personnel. The study team included experienced female, doctoral level researchers/clinicians/trainers, and a master’s level bicultural, bilingual research associate, who were actively involved in all phases of the study. Procedures were reviewed by the University Institutional Review Board and the City Institutional Review Board and were determined to be exempt, as they did not meet the federal criteria for human subjects research.

The adaptation process was iterative and dynamic, with concurrent activities informing each other (50). Acceptability was defined as the perception that ParentingWell content is appealing and useful to potential practitioners and persons served, determined through discussion with community context experts participating on the Adaptation Team (33, 50). Fit reflected the extent to which ParentingWell was viewed or modified to be relevant to the local context with input from community representatives. Feasibility was defined through the evaluation of the process of implementing training for ParentingWell in the new service context via the practitioner Learning Collaborative.

Descriptive data were provided by adaptation process participants. Meetings, resource reviews, training sessions, and feedback were documented with detailed, verbatim notes and summaries, available for systematic review. Community reviewer feedback contributed to the compilation of the adapted ParentingWell Practice Profile and resources. Learning Collaborative participants provided responses to brief surveys regarding satisfaction and suggestions for future implementation. Modifications were made to ParentingWell Learning Collaborative procedures and materials as challenges emerged and feedback became available.

### 2.1 The adaptation team: context

The Adaptation Team (AT) was a diverse, multi-disciplinary team of policymakers and practitioners, with backgrounds and expertise in behavioral health, psychiatric rehabilitation, maternal-child health, parenting, policymaking, program development, integrated care, provider training, public health, and research, and extensive knowledge of Philadelphia’s behavioral health system and persons served. Participants represented the City of Philadelphia’s Department of Behavioral Health and Intellectual disAbility Services (DBHIDS), Community Behavioral Health (CBH), and the Health Federation of Philadelphia (HFP). DBHIDS functions as a single-payer public health system using federal, state, and local dollars, including Medicaid, to oversee behavioral health care, intellectual disability supports, and early intervention services for children, adults, and families.

CBH is a nonprofit behavioral health managed care organization (MCO), operating as a non-profit 501(c) (3) corporation created and contracted by DBHIDS to manage the administration of behavioral health Medicaid benefits for more than 800,000 residents, half of the City’s population. CBH provides care coordination, manages a network of over 200 behavioral health providers, and authorizes payment for care. The Health Federation of Philadelphia supports Community Health Centers, as well as other organizations that deliver healthcare to vulnerable individuals through advocacy, professional and program development, and consultation. HFP’s programing includes a focus on families impacted by parental behavioral health conditions. Adaptation Team members had been or were currently providing behavioral health services across all three organizations.

The AT guided and oversaw the following: (1) planning for and implementing ParentingWell and the pilot ParentingWell Learning Collaborative, especially designed to meet the needs of the Philadelphia stakeholder community; (2) the collection of feedback on existing ParentingWell materials to guide adaptation (including the identification of stakeholders to provide feedback, the specification of materials for stakeholders to review, the specification of feedback collection methods, and the review of findings); and (3) the identification of stakeholders who would benefit from participation in the ParentingWell Learning Collaborative, and recommendations for training implementation.

### 2.2 Community stakeholder resource review: content

The Adaptation Team created eight packets of materials for community stakeholder resource review to tailor the ParentingWell approach and content to the new target population. Two packets were prepared for each of the following ParentingWell core elements: Engage, Explore, & Plan; one packet was prepared for the core element of Access and Advocate. One additional packet contained ParentingWell self-assessment and supervisory tools. Each packet contained an overview of ParentingWell that was identical across packets and was approximately two pages in length. Each packet also contained an overview of its core element, and sample exercises/activities to correspond with the core element (i.e., an activity to identify potential sources of back-up childcare within the Plan packet). The core elements with two packets had different activities in each version of the packet, so that more activities were reviewed by community stakeholders. Each packet was approximately ten pages long.

Adaptation Team members recruited community stakeholders to complete material review. Community stakeholders included peer specialists, peer specialist supervisors, and representatives of diverse government and community organizations such as the Philadelphia Coalition, the Latino Behavioral Health Coalition, the Alliance of Community Service Providers, the Coalition of Culturally Competent Providers, the Philadelphia Department of Human Services, and many others. Community stakeholders were contacted directly by Adaptation Team members, who were members of their professional networks.

After learning about the project and agreeing to participate, community stakeholders provided feedback on the packets they reviewed via an online survey. The survey was identical across packets. Respondents identified the title of the packet they were reviewing. The survey included open-ended questions such as: (1) Do you think the content you read would be useful for parents and service providers in your community? Please explain. (2) What is helpful about the content that you read? How could it be helpful to service providers and/or parents in your community? (3) What is not helpful about what you read? Or, how can we improve the content that you read to make it more helpful to service providers and/or parents in your community? And (4), Do you have any other feedback that you would like to share? These questions were designed to elicit feedback that could potentially span many types of content modifications (i.e., tweaking, deleting, lengthening, shortening, repackaging, etc.) (48). Reviewers received a gift card for completing review of a packet.

### 2.3 The ParentingWell learning collaborative: training

#### 2.3.1 The PWLC application process

All procedures were reviewed and managed by the Adaptation Team. A Request for Applications (RFA) to join the ParentingWell Learning Collaborative was issued by Philadelphia Community Behavioral Health (CBH) in May 2023. The RFA provided background information and a description of ParentingWell, proposal and submission requirements. The application process was open to agencies in good standing with CBH, currently enrolled in Medicaid/Medicare programs, and appropriately licensed. Expectations for participants were outlined, including that participating agencies identify a team of up to four adult-serving staff volunteers, at least one of whom must be a supervisor or program manager responsible for leading implementation at the selected site. Designated staff had to be able to identify parents or adults who are planning to become parents with whom they were working. Agencies were required to identify a senior leader or executive sponsor for the initiative.

Participating in the PWLC was proposed to involve attending an orientation meeting, four to six virtual training sessions (approximately two hours each), monthly virtual coaching sessions for four months following the conclusion of training, one virtual debriefing session, and access to a BaseCamp virtual information hub. A virtual information session was provided to potential applicants, with the opportunity to ask questions of Philadelphia CBH and ParentingWell staff. Applications were received and reviewed by CBH and ParentingWell staff. One provider agency and staff from the CBH Community Based Care Management primary care and perinatal teams were approved to participate. Ten continuing education credits were available to social worker participants, approved by the Philadelphia chapter of the National Association of Social Workers.

#### 2.3.2 The orientation sessions

Two orientation sessions were held in November 2023 with PWLC participants, one in-person and one virtually. Participants were provided a brief presentation with the opportunity to ask questions about the Learning Collaborative process and expectations. Copies of the ParentingWell Practice Profile and Workbook were made available. Participants completed a brief background and demographic survey, and a survey regarding current practice with parents. See **Supplementary Material**: the ParentingWell Practice Survey.

#### 2.3.3 ParentingWell learning collaborative sessions

Four two-hour virtual learning collaborative sessions were held in November and December 2023. The sessions were led by trained, experienced ParentingWell team members, one of whom was bicultural and Spanish-fluent. Each session focused on one of the four core elements of the ParentingWell Practice Profile: Engage, Explore, Plan, and Access and Advocate. Sessions began with a welcome and opening interactive activity. An agenda for the day’s session was provided with objectives, along with references to resources available in the BaseCamp virtual hub and ParentingWell Workbook. Large group information sessions were followed by small group interactive activities targeting the application of information and skill development. Brief breaks were scheduled mid-session. Sessions included relevant videos and audio clips. ParentingWell team members provided a wrap-up summary, overview of next steps, and suggested homework assignments, for participants to apply information and skills in their practice settings. Agendas, slides, and resources were provided in English and Spanish. Satisfaction surveys were completed at the end of each PWLC session to solicit feedback and inform PWLC improvements.

#### 2.3.4 Coaching and debriefing

Four one-hour coaching sessions were offered in February and March 2024, facilitated by the ParentingWell team, and focused on ParentingWell core elements as they related to participants’ experiences. Participants were encouraged to bring examples of positive as well as challenging interactions with parents and families to the coaching sessions for discussion with the ParentingWell team and practice colleagues. A follow-up debriefing session was offered but determined by participants to be unnecessary.

## 3 Results

The iterative intervention adaptation process provided opportunity to assess ParentingWell acceptability, through the efforts of context experts on the Adaptation Team; fit, through content review by community stakeholders; and feasibility, through the implementation of the ParentingWell Learning Collaborative to train local practitioners.

### 3.1 The adaptation team: acceptability

Adaptation Team members (*n* = 8) represented different community organizations or divisions within Philadelphia DBHIS, along with ParentingWell purveyors. All were female, 50% were White, with graduate level education, whose professions included health and human services, psychology, human development, social work and social welfare. (See **Table 1**). The AT met twenty times between October 2021 and June 2023. Each meeting included between 3 and 7 Philadelphia stakeholders (in addition to 2 to 3 team members representing ParentingWell purveyors). The Adaptation Team made recommendations for ParentingWell implementation and assessment, based on their extensive knowledge of the community context and target population. The Team facilitated connections to ParentingWell content reviewers, drawing from their experience with the community and as service users, including parent peer specialists and the DBHIDS Family Member Committee, which was under Philadelphia’s System of Care and included parents and caregivers with behavioral health conditions, and/or who had children with behavioral health conditions or child welfare involvement.

**Table 1:** Demographic characteristics of participants.

Characteristics	Adaptation core team (N = 8)^a^		Community resource reviewers (N = 18)		PW learning collaborative participants^b^ (N = 15)	
	N	%	N	%	N	%
Age						
22-34	1	12	2	11	5	33
35-44	2	25	7	39	4	27
45-54	3	38	5	28	3	20
55-64+	2	25	3	16	3	20
Choose not to disclose	0	0	1	6	0	0
Gender						
Female	8	100	18	100	10	67
Male	0	0	0	0	5	33
Latino(a)	1	13	6	33	7	47
Race						
White	4	50	5	28	9	60
Black or African American	2	25	9	50	2	13
American Indian/Alaska Native	0	0	1	6	0	0
Bi-racial	1	13	0	0	0	0
Choose not to disclose	1	13	3	17	4	27
Highest level of education						
High school graduate or the equivalent	0	0	0	0	1	7
Partial college credit	0	0	3	17	1	7
Associate’s degree/Bachelor’s degree	0	0	4	22	3	20
Master’s degree/Doctoral degree (e.g., PhD, EdD)/Professional degree	8	100	11	61	10	67
Discipline for advanced degree						
Health and Human Services Professions	3	38	6	33	2	14
Psychology and Human Development	3	38	5	28	3	20
Social Work, and Social Welfare	2	25	3	17	5	33
Other	0	0	0	0	2	14
N/A	0	0	2	11	1	7
Blank	0	0	2	11	2	13

a Percentage not always equal exactly to 100 due to rounding.

b Of the 23 total participants listed across 4 sessions, 15 participants completed the background survey.

To cast a wider net for ParentingWell Learning Collaborative participation, the AT recommended using a Request for Applications (RFA) process rather than targeting specific providers for inclusion. The RFA detailed time commitment, expectations, incentives for participation and the need to complete all requirements to earn the incentives. All professional disciplines were welcomed from psychologists, to social workers to peer specialists. The AT recommended in-person orientation sessions where possible, followed by virtual training sessions. They stressed the importance of providing CEUs for individual practitioners, and financial incentives for the agencies with staff participants, as training is not a reimbursable activity. These were considered essential in enhancing the acceptability of the PWLC and ParentingWell practice.

### 3.2 Community stakeholder resource review: fit

Community stakeholders (*n* = 18) provided detailed review of ParentingWell resources. (See **Table 1**). They were spread across age ranges, all female, one-third were Latina and half were Black. Many had some college education, with over 60% having a graduate degree. They identified as health and human services, psychology, human development, social work or social welfare professionals.

Stakeholders consistently answered “yes” to the question: Do you think the content you read would be useful for parents and service providers in your community? When asked to explain, stakeholders identified several helpful elements related to the content, including its relevance and usefulness. Comments regarding relevance included: “This content is appropriate for the community”; “I work with many families who have experienced trauma. This seems to be a trauma-informed approach, using strengths of the family”; and “The parents will be getting much needed information and resources.” Regarding usefulness, one stakeholder shared, “It gives a clear outline of how to interact with the parent. Questions to ask and questions they should steer away from.” Another shared, “The materials provide a blueprint for clinicians to engage and ask questions about parenting and family life, without coming across as too clinical.” Some stakeholders shared personal reactions, e.g., “As a parent who is coping with mental illness, I think that if this program had been available sooner, I would have found stability and success in my parenting, daily life, career, etc., a long time ago”.

Stakeholders shared suggestions for improvement related to accessibility and cultural/contextual relevance. One stakeholder shared, “ I would like to see the terminology broken down in language that would be easy for any parent on any level to understand.” Other comments related to accessibility include, “I believe a visual (picture), in between the subheading might be helpful” and “The homework/activities take into account that there 5are individuals in the community with reading and comprehension issues”.

In response to this feedback, we developed a plain language version of the ParentingWell Workbook, in both English and Spanish. We also developed a plain language version of the Practice Profile Executive Summary.

### 3.3 The ParentingWell learning collaborative: feasibility

While 23 participants attended Learning Collaborative sessions over time, only a subset completed the background and demographics survey (*n* = 15) (see **Table 1**). They were evenly split across age categories. Two-thirds were female and nearly half were Latino/a. Sixty percent were White, several were Black, and over one-quarter chose not to disclose. The majority had some college credit or degree, or a graduate degree. Of those completing the background and demographics survey, the majority identified as health and human services, psychology, human development, social work, or social welfare professionals.

Twenty-two Learning Collaborative participants complete the ParentingWell Practice Survey prior to attending training sessions. This survey includes 44 Likert-type items (“1 = strongly disagree” to “7 = strongly agree”), developed to reflect four components of the Theory of Planned Behavior that may predict practice behavior change (45). These include, for example: (1) “Talking with persons served about mental health and parenting is supportive to their recovery” (attitudes); (2) “My agency has clear policies and procedures for working with adults who are or hope to become parents” (subjective norms); (3) “My training and experience provide a solid base of skills for working together with parents”(perceived behavioral control); and (4) I expect to find ways to identify and address the needs of people who are parents” (intention to change behavior). (See **Supplementary Material** – ParentingWell Practice Survey.) In general, participants agreed that talking with an adult person served about parenting and family life is important for the parent and rewarding for the practitioner. Agencies and supervisors were described as supportive of practitioners who talked with adults about parenting and family life. While practitioners indicated they had a solid skill base for working together with parents, they wanted to identify ways to be more sensitive to the parenting and family experiences of persons served.

Attendance over the four training sessions ranged from 6 to 13 active participants. The satisfaction survey (administered at the end of each virtual learning collaborative session) consisted of six Likert-type items reflecting satisfaction with the format, content, and training provided, rated on a scale from “1 = strongly disagree” to “7 = strongly agree” (see **Table 2**). The mean rating for each session was about 5.5, indicating general satisfaction with the training sessions. Though the range of responses for each item was wide, the majority of ratings for each session fell in the 5 to 7 range. The survey also included the following three open-ended questions: (1) What did you like about today’s session? (2) What did you learn in today’s session? (3) What would you change about today’s session? Participants provided positive feedback pertaining to the structure (i.e., “it was interactive”, and “large group format discussion was helpful”); the content and resources provided (i.e., “the questions and activities were thought provoking”); and the group activities and dynamics (i.e., “I liked hearing about everyone’s different cultural practices when they spoke about what they did on Thanksgiving.”). Participants identified several topics that they learned about, including self-care, self-reflection, and “developing better ways to engage parents”.

**Table 2:** ParentingWell learning collaborative participant satisfaction ratings across four sessions.

Session evaluation statements^a^	Mean^b^	Range
I am satisfied with the format of today’s PW session.	5.5	2-7
I found this PW session to be applicable to my role.	5.4	2-7
I am satisfied with the trainer(s) who led today’s PW session.	5.7	2-7
I am satisfied with my overall experience at today’s PW session.	5.5	2-7
The balance between presentations, discussion and activities fits my style of learning.	5.4	1-7
I would recommend this PW session to other behavioral health practitioners.	5.5	2-7

a Attendance in sessions ranged from 6 to 13 participants.

b Likert-type items were rated on a scale from “1 = strongly disagree” to “7 = strongly agree”.

With regard to the third question pertaining to suggestions for improvement, participants’ responses reflected diverse opinions and perspectives. One participant suggested “add ice breakers”, while another noted the following critique: “Spending a lot of time on what seems like ice breakers rather than the applicable questions to ask parents”. A third person suggested, “Describe why you’re asking a question that looks like an ice breaker to help group understand why or what we’re learning.” Similarly, some people identified breakout rooms for smaller group discussion as a helpful component of the training, while one person said, “Breakout rooms feel unnecessary/too long.” Taken together, the feedback conveys a diversity of preferences. It may be possible to address seemingly disparate concerns with the provision of additional information setting the stage or providing the rationale for discussion. For example, breakout rooms or ice breakers might not seem redundant if additional instructions or context is provided.

Coaching sessions provided additional opportunity for the elaboration of training themes and feedback on training content and format. During coaching sessions, participants reiterated that their agency has been and continues to be family-focused. For example, one participant shared that all staff visit the homes of their clients. Despite this baseline level of familiarity with family-focused practice (as identified via the baseline ParentingWell Practice Survey, described earlier), participants expressed that they benefitted from participation in the Learning Collaborative. One participant shared, “Feedback is always constructive,” and another participant reported receiving reinforcement that “you are not alone with a case”. Notably, feedback pertaining to the virtual/Zoom format was largely neutral or negative (i.e., “I don’t like Zoom; I like to do it in person,” and “The good thing about Zoom is time. You don’t have to drive. But on the other hand, you get distracted”).

## 4 Discussion

This paper describes the adaptation process as the first step in implementing and sustaining ParentingWell in the Philadelphia behavioral health community, which represents a new, diverse target population and urban setting. Multiple adaptation activities and processes (i.e., engagement with an Adaptation Team, resource content review, and the ParentingWell Learning Collaborative) with diverse stakeholders provide preliminary support for the acceptability, fit, and feasibility of the approach within the new community context.

Additionally, the adaptation process illuminated essential considerations related to the adaptation of ParentingWell. First, our adaptation process was designed to incorporate project champions, who are personnel within CBH, the corporation that manages the administration of behavioral health Medicaid benefits for more than 800,000 Philadelphia residents. These champions were key points of contact throughout the project; all activities reflected their vision and input. Our inclusion of champions at each substantive activity and decision-making point reflects the importance of champions for successful program implementation in health care (51). For example, as part of the Adaptation Team, champions recommended the format for the content review, identified and recruited relevant agencies for participation in the Learning Collaborative, and advised on key decisions regarding the Learning Collaborative (i.e., virtual rather than in-person format). While the contributions of the AT were instrumental, the project was undoubtedly influenced when multiple champions left their positions of employment while the project was in progress.

Given the high rate of turnover within the public behavioral health sector (52), successful adaptation and, ultimately, implementation, testing, and sustainability likely include strategies to mitigate the impact of staff turnover. This is especially relevant given the importance of relationships within implementation efforts (47). Relevant strategies may include strategies for efficient timelines for implementation and research, so that staffing can be as consistent as possible, without sacrificing comprehensiveness or rigor. A second strategy may include having a contingency plan in place in the event of staff turnover. Given the challenges of high staff turnover in public behavioral health systems, with the potential impact on persons served, it is important to note that ParentingWell resources include an Individual Skill Development Plan and ParentingWell Self-Assessment – Guidance for Supervisors, available on the ParentingWell website (https://heller.brandeis.edu/parents-with-disabilities/support/parenting-well.html). These resources may be particularly useful in the agency context, for ongoing training, supervision, and support of continuing and new staff members.

Other considerations reflect the extent to which flexibility can be a crucial component of successful adaptation and implementation. Learning Collaborative participants, via the ParentingWell Practice Survey administered before the Collaborative began, indicated they had a solid skill base for working together with parents. They were family-focused providers, unlike the participants in the Massachusetts iteration, who had been adult mental health service providers and not experienced or adept at considering clients’ family roles. Thus, Philadelphia participants were eager to explore concrete tools for working with parents, and were perhaps less interested in background information about why it is important to incorporate conversations about parenting into routine practice. Also, nearly half of the participants were Latino/a. The project team included a bilingual and bicultural staff member who translated materials and instructions. She also provided guidance on cultural norms within educational and training contexts, such as preferences for instructor-led lectures and assignments, rather than interactive conversations and activities. These considerations were crucial for the successful implementation of the Learning Collaborative. We didn’t learn these things about participants until relatively late in the adaptation process (i.e., after materials had been updated based on feedback from content reviewers). In future iterations, it might be helpful to start the Learning Collaborative with a conversation around mutual expectations (participants and project staff) for engagement, taking community and cultural context, characteristics, and preferences into account. Overall, this speaks to the importance of flexibly integrating feedback and addressing contextual considerations throughout the adaptation process – this is not limited to a single process component.

Finally, as noted in the results section, feedback pertaining to the virtual format of the Learning Collaborative (Zoom) was largely negative. Additionally, there was minimal (if any) engagement on Base Camp, the virtual hub for sharing resources and experiences. The Learning Collaborative participants from the Massachusetts iteration used Base Camp extensively (45). We selected the virtual format in Philadelphia in response to input from the Adaptation Team, who suggested that the virtual format would mitigate participant challenges in scheduling, travel time, and parking costs. However, the needs and preferences of the specific participating agencies were unique. Learning Collaborative participants were not uniformly satisfied with the virtual approach, which likely influenced their engagement. Thus, contextual considerations were not uniform across the Philadelphia behavioral health community. Our experience underscores the notion that flexibility and individualization are required throughout the adaptation and learning collaborative/coaching process. Hybrid or tailored, organization-specific models may be more engaging. When contemplating longer-term initiatives, like establishing a learning collaborative or providing ongoing coaching opportunities to mitigate staff turnover and support implementation fidelity, the best, most efficient use of staff time must be considered and balanced with the benefits of virtual, in person, or hybrid models, and ultimate impact on parents and families served. The identification of a long-term training organization or “home” could also serve as a resource to support ongoing training, coaching, and supervision efforts, with potential certification guidelines to promote family-focused practice and ParentingWell training and use.

### 4.1 Limitations

While we conducted follow-up coaching sessions several months after the ParentingWell Learning Collaborative, our contact with participants ended at the conclusion of these sessions. Therefore, we do not know how many of these participants still work at these agencies, and if or how supervisors facilitate ongoing use of ParentingWell within the organizational context given the likelihood of staff turnover. Longitudinal research could assess and address this issue, as well as many others (e.g., ultimate impact on parents, children, and families).

### 4.2 Future directions

Considerations for future directions broadly relate to at least two categories: considerations related to comparable and ongoing adaptation projects, and considerations for work that is yet to be done within the ParentingWell adaptation and implementation processes. For similar adaptation endeavors, future research should explore the complex intersections of contextual and cultural considerations when adapting an intervention in a new community and/or with a new target population. For example, as noted previously, ParentingWell Learning Collaborative participants had a high level of baseline skills pertaining to family-focused practice, *and* participants had differing levels of comfort with interactive training exercises. Optimal methods to differentiate instruction to meet the needs of more or less skilled participants may partially depend on participants’ levels of involvement (i.e., some methods for differentiating instruction may rely on high levels of interaction). These methods may also look different in virtual vs. in person formats, which comprises an additional consideration.

Regarding next steps in adapting ParentingWell, future research should include longitudinal data collection with both providers and parents to ascertain the extent to which providers employ ParentingWell family-focused tools and strategies, and parent and child outcomes are improved (53). This will involve the development of assessment strategies for implementation fidelity. Outcome measurement approaches for provider training and ParentingWell implementation should include both self-report and observational measures, when possible. Parents and families, the ultimate service users, should inform the implementation process, research methods, and selection of outcomes to ensure relevance and enhance the likelihood of positive impact. Ultimately, rigorous longitudinal research should measure the impact of ParentingWell on parents served and their children. This will provide insight into the most important aspect of ParentingWell: its potential to improve outcomes for parents with mental illness and their families.

## Data Availability

The datasets presented in this article are not readily available. Given the qualitative nature of the data and the concern for the privacy of participants, many of whom are agency leaders, data are held by the authors. Requests to access the datasets should be directed to jnicholson@brandeis.edu.
