# Supplementary material for: ParentingWell: adapting a family-focused practice for parents with mental illness
Source: Front Psychiatry. 2025 Sep 19;16:1678134. doi: 10.3389/fpsyt.2025.1678134 (PMC12491307; doi:10.3389/fpsyt.2025.1678134)
Supplement: Supplementary file 1 [file Table1.docx]

Supplementary Material

**ParentingWell: Adapting a Family-Focused Practice**

**for Parents with Mental Illness**

# The ParentingWell® Practice Survey

The ParentingWell® Practice Survey, drawn from the Theory of Planned Behavior, provides for useful pre- and post-assessment of training and coaching in family-focused practice approaches for service providers working together with adults who are parents living with mental health conditions.

**Instructions:** Please circle the answer that best describes your thoughts or opinions about each item. A note about language - “people” or “individuals” or “person served” refers to any adult with whom you are working, who is living with a mental health condition/behavioral health challenge, and who is a parent or is planning to become a parent.

| **Not applicable** | **Strongly**  **Disagree** | **Disagree** | **Slightly**  **Disagree** | **Neither agree or disagree** | **Slightly**  **Agree** | **Agree** | **Strongly**  **agree** |
| --- | --- | --- | --- | --- | --- | --- | --- |
| **N/A** | **1** | **2** | **3** | **4** | **5** | **6** | **7** |

| 1. My agency has clear policies and procedures for working with adults who are or hope to become parents. | N/A | 1 | 2 | 3 | 4 | 5 | 6 | 7 |
| --- | --- | --- | --- | --- | --- | --- | --- | --- |
| 1. Workers without experience as parents are not equipped to work with people who are parents. | N/A | 1 | 2 | 3 | 4 | 5 | 6 | 7 |
| 1. I expect to find ways to identify and address the needs of people who are parents. | N/A | 1 | 2 | 3 | 4 | 5 | 6 | 7 |
| 1. I want to review my work routine to identify ways to be more sensitive to the parenting and family experiences of persons served. | N/A | 1 | 2 | 3 | 4 | 5 | 6 | 7 |
| 1. I am not interested in working with individuals with mental health conditions on their parenting issues. | N/A | 1 | 2 | 3 | 4 | 5 | 6 | 7 |
| 1. My training and experience provide a solid base of skills for working together with parents. | N/A | 1 | 2 | 3 | 4 | 5 | 6 | 7 |
| 1. Talking with adults I serve about their parenting and family experiences is not in my job description. | N/A | 1 | 2 | 3 | 4 | 5 | 6 | 7 |
| 1. I am confident working with people on their issues as parents. | N/A | 1 | 2 | 3 | 4 | 5 | 6 | 7 |
| 1. I don’t have anyone to turn to for advice when the people I serve want to talk about parenting and family life. | N/A | 1 | 2 | 3 | 4 | 5 | 6 | 7 |
| 1. The individuals I work with would rather not talk about themselves as parents, about their children or their families. | N/A | 1 | 2 | 3 | 4 | 5 | 6 | 7 |
| 1. I can’t imagine myself talking with people about their experiences as parents. | N/A | 1 | 2 | 3 | 4 | 5 | 6 | 7 |
| 1. Talking with adults about parenting and family life will help them interact in more positive ways with children and family members. | N/A | 1 | 2 | 3 | 4 | 5 | 6 | 7 |
| 1. My agency is not mandated to address the issues of person served as parents. | N/A | 1 | 2 | 3 | 4 | 5 | 6 | 7 |
| 1. The adults I work with expect me to ask them about parenting and family life. | N/A | 1 | 2 | 3 | 4 | 5 | 6 | 7 |
| 1. I do not have the skills for talking with people about their role and responsibilities as parents and family members. | N/A | 1 | 2 | 3 | 4 | 5 | 6 | 7 |
| 1. My agency would have to make some changes in policies and procedures to support working with adults as parents. | N/A | 1 | 2 | 3 | 4 | 5 | 6 | 7 |
| 1. My supervisor supports me in talking with adults about parenting and family life. | N/A | 1 | 2 | 3 | 4 | 5 | 6 | 7 |
| 1. I am not inclined to make changes in my practice that would be required to identify and address the issues of adults who are parents. | N/A | 1 | 2 | 3 | 4 | 5 | 6 | 7 |
| 1. There are times when I am at a loss in talking with individuals about their parenting and family life experiences. | N/A | 1 | 2 | 3 | 4 | 5 | 6 | 7 |
| 1. Thinking and talking about my own family experiences informs the work I do with parents or those who want to be parents. | N/A | 1 | 2 | 3 | 4 | 5 | 6 | 7 |
| 1. Talking with persons served about the typical challenges of parenting and family life makes them feel inadequate and alone. | N/A | 1 | 2 | 3 | 4 | 5 | 6 | 7 |
| 1. Thinking and talking about parenting and family experiences with persons served is expected in my workplace. | N/A | 1 | 2 | 3 | 4 | 5 | 6 | 7 |
| 1. I intend to become better able to help adults talk about their parenting and family experiences. | N/A | 1 | 2 | 3 | 4 | 5 | 6 | 7 |
| 1. Talking with adults about parenting and family life makes them more anxious about any mistakes they are making. | N/A | 1 | 2 | 3 | 4 | 5 | 6 | 7 |
| 1. It’s ok for me to talk about anything a person wants to talk about, including parenting and family life. | N/A | 1 | 2 | 3 | 4 | 5 | 6 | 7 |
| 1. Encouraging parents to reflect on their family experiences confuses and upsets them, and undermines their ability to cope with crises. | N/A | 1 | 2 | 3 | 4 | 5 | 6 | 7 |
| 1. Talking with persons served about mental health and parenting is supportive to their recovery. | N/A | 1 | 2 | 3 | 4 | 5 | 6 | 7 |
|  |  |  |  |  |  |  |  |  |

**Instructions:** For the following items, please circle the number that shows where on the continuum you fall, between the two ends provided.

| **Talking with an adult person served about parenting and family life is...** | | | | | | | | | | | | | | | | |
| --- | --- | --- | --- | --- | --- | --- | --- | --- | --- | --- | --- | --- | --- | --- | --- | --- |
| 1. Important | 1 | 2 | 3 | 4 | | 5 | | 6 | | 7 | | Not important | | | | |
| 1. Beneficial (for them) | 1 | 2 | 3 | 4 | | 5 | | 6 | | 7 | | Harmful (for them) | | | | |
| 1. Uncomfortable (for me) | 1 | 2 | 3 | 4 | | 5 | | 6 | | 7 | | Comfortable (for me) | | | | |
| 1. Motivating (for them) | 1 | 2 | 3 | 4 | | 5 | | 6 | | 7 | | Disheartening (for them) | | | | |
| 1. Not satisfying (for me) | 1 | 2 | 3 | 4 | | 5 | | 6 | | 7 | | Rewarding (for me) | | | | |
| **Extremely Unlikely 🡪🡪🡪🡪🡪🡪 Extremely Likely** | | | | | | | | | | | | | | | | |
| 1. If I talk with a person served about parenting, I know I am doing something positive for that person. | | | | | N/A | | 1 | | 2 | | 3 | | 4 | 5 | 6 | 7 |
| 1. It causes a lot of worry and concern for the adult if I talk about parenting. | | | | | N/A | | 1 | | 2 | | 3 | | 4 | 5 | 6 | 7 |
| 1. If I ask about parenting, I am likely to uncover problems. | | | | | N/A | | 1 | | 2 | | 3 | | 4 | 5 | 6 | 7 |
| 1. If I talk about parenting, it will take more time. | | | | | N/A | | 1 | | 2 | | 3 | | 4 | 5 | 6 | 7 |
| **Extremely Undesirable🡪🡪🡪🡪🡪🡪Extremely Desirable** | | | | | | | | | | | | | | | | |
| 1. Doing something positive for the person served as a parent is… | | | | | N/A | | 1 | | 2 | | 3 | | 4 | 5 | 6 | 7 |
| 1. Causing a lot of worry and concern about parenting for the person served is… | | | | | N/A | | 1 | | 2 | | 3 | | 4 | 5 | 6 | 7 |
| 1. Uncovering problems in parenting is… | | | | | N/A | | 1 | | 2 | | 3 | | 4 | 5 | 6 | 7 |
| 1. Having to take more time with adults served who are parents is… | | | | | N/A | | 1 | | 2 | | 3 | | 4 | 5 | 6 | 7 |

| **Not applicable** | **Strongly**  **Disagree** | **Disagree** | **Slightly**  **Disagree** | **Neither agree or disagree** | **Slightly**  **Agree** | **Agree** | **Strongly**  **agree** |
| --- | --- | --- | --- | --- | --- | --- | --- |
| **N/A** | **1** | **2** | **3** | **4** | **5** | **6** | **7** |

| 1. I regularly ask persons served about parenting, their family circumstances and experiences. | N/A | 1 | 2 | 3 | 4 | 5 | 6 | 7 |
| --- | --- | --- | --- | --- | --- | --- | --- | --- |
| 1. I routinely work with persons served to set goals related to parenting and family life. | N/A | 1 | 2 | 3 | 4 | 5 | 6 | 7 |
| 1. Together we regularly monitor the person’s progress in achieving parenting and family goals. | N/A | 1 | 2 | 3 | 4 | 5 | 6 | 7 |
| 1. I routinely assist the person served in accessing additional resources – personal and professional – to support them in their parenting role and family life. | N/A | 1 | 2 | 3 | 4 | 5 | 6 | 7 |

**Thank you for taking the time to complete this survey.**
